# Significant resolution of multiple adult-onset xanthogranulomas with MEK inhibitor therapy

**DOI:** 10.1016/j.jdcr.2026.06.068

**Published:** 2026-07-11

**Authors:** Xin Wang, Emily R. Nadelmann, Hailey Konisky, Paul Chu, Eli L. Diamond, Tobi Klar

**Affiliations:** a Division of Dermatology, Department of Medicine, Montefiore Medical Center/Albert Einstein College of Medicine, Bronx, New York; b Department of Neurology, Memorial Sloan Kettering Cancer Center, New York, New York

**Keywords:** multiple adult-onset xanthogranulomas, MAXG, non-Langerhans cell histiocytosis, MEK inhibitor, KRAS mutation

## Introduction

Xanthogranulomas are non-Langerhans cell histiocytoses that present as lipid-filled cutaneous lesions. The Histiocyte Society classifies histiocytoses and neoplasms of macrophage-dendritic cell lineages into 5 groups based on clinical, pathological, radiological, and molecular features: Langerhans-related (L-group), cutaneous/mucocutaneous (C-group), malignant (M-group), Rosai-Dorfman disease (R-group), and hemophagocytic lymphohistiocytosis (H-group). Xanthogranulomas are categorized as C-group histiocytoses.^1^^,^^2^ Treatment varies widely and includes topical corticosteroids, retinoids, and antimetabolites.^3^ We present a refractory case of adult-onset xanthogranulomas (AXG) with a somatic KRAS mutation activating the MAPK pathway. MEK inhibitor treatment led to remarkable improvement, highlighting its potential role in AXG management.

## Case presentation

**Fig 1:**
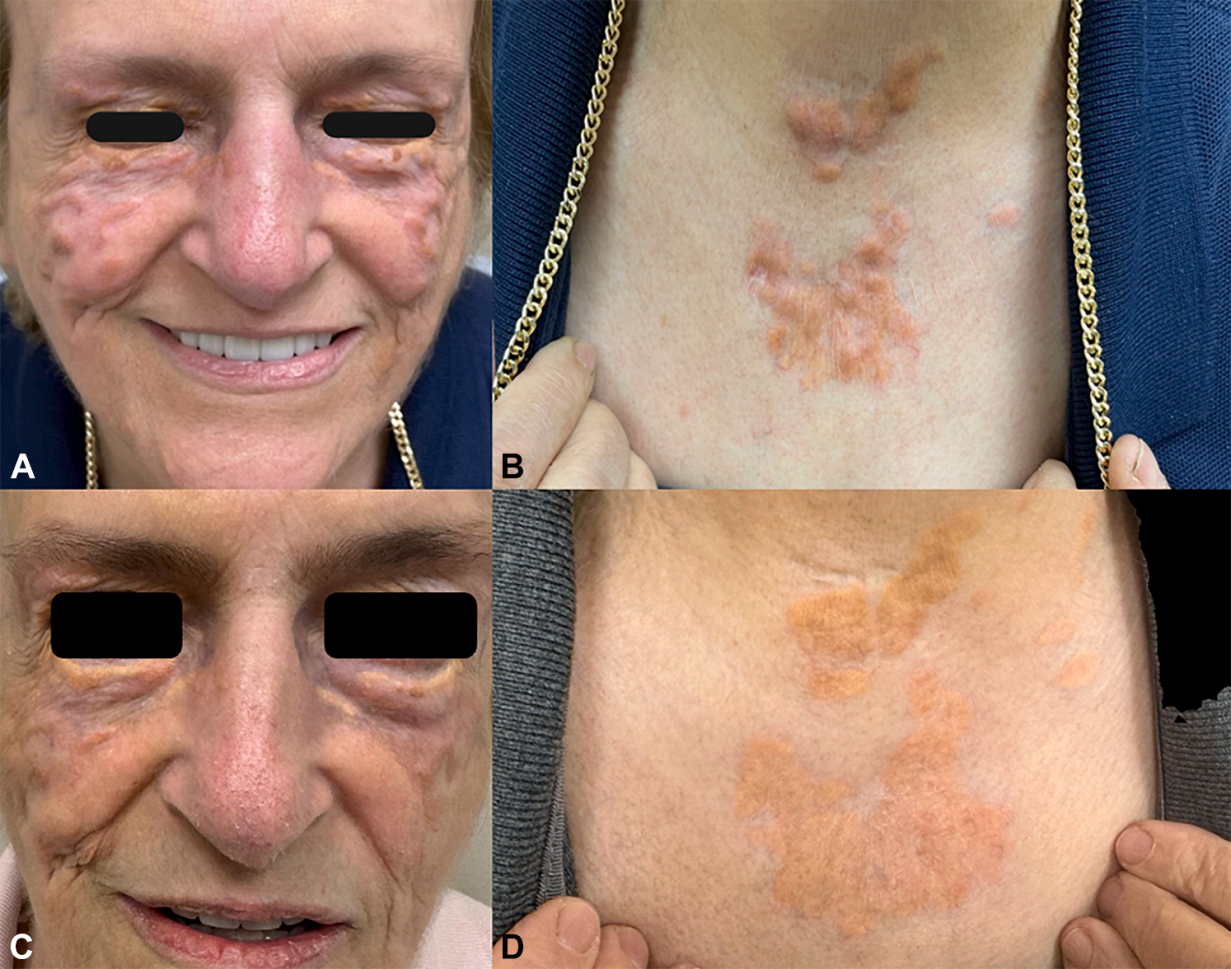
Initial clinical presentation. **A,** Yellow-to-skin-colored dermal plaques are observed on the upper and lower eyelids. The bilateral cheeks display erythematous-to-brown firm dermal plaques. **B,** Erythematous, yellow, and brown thickened dermal plaques are also visible on the anterior neck and chest. 2 months status post-MEK inhibitor/intralesional triamcinolone treatment. **C,** Facial lesions have less visible improvement but flattened dermal plaques. **D,** Lesions on the chest demonstrates marked improvement, flattening of lesions, and reduction of erythema.

**Fig 2:**
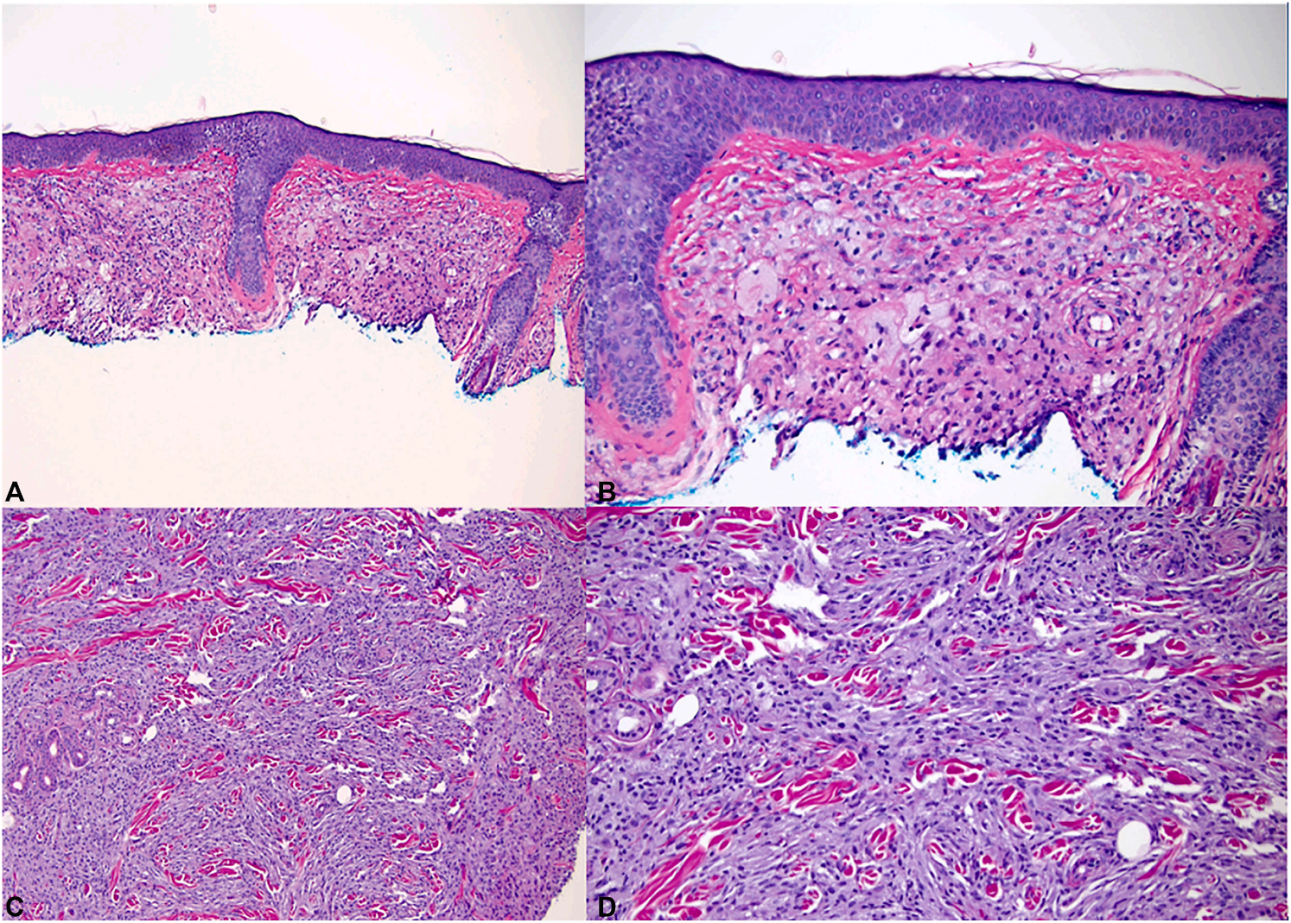
Histopathology of xanthogranuloma. **A** and **B,** The dermis showed a dense infiltrate of foamy histiocytes, Touton giant cells, and sparse lymphocytes (40× original magnification, 100× Hematoxylin & Eosin). Histopathology of xanthogranulomatous dermatitis. **C** and **D,** The biopsy shows a dense dermal collection of eosinophilic and foamy histiocytes (40× original magnification, 100× Hematoxylin & Eosin).

A 74-year-old female with hypercholesterolemia, asthma, and hypertension presented with several years of asymptomatic eyelid and facial lesions. Examination revealed more than 10 yellow-to-skin-colored plaques on the bilateral upper eyelids and multiple well-demarcated erythematous-to-brown plaques on the malar cheeks (Fig 1, *A*). Biopsy of a left malar plaque demonstrated foamy histiocytes, Touton giant cells, and sparse lymphocytes, consistent with xanthogranulomatous dermatitis (Fig 2, *A* and *B*). Immunohistochemistry was negative for BRAF V600E.

Eight months following the initial visit, additional erythematous-to-yellow-brown plaques appeared on the chest and progressively coalesced into a 7.1-cm, irregular plaque (Fig 1, *B*). Histopathology demonstrated abundant foamy histiocytes, consistent with xanthogranulomatous dermatitis (Fig 2, *C* and *D*). With more than 5 papular/nodular lesions and histology consistent with xanthogranulomas, a diagnosis of multiple adult-onset xanthogranulomas (MAXG) was established.

Given the association between AXG and hematologic malignancies, annual evaluation including complete blood count, serum protein electrophoresis, and full-body positron emission tomography/computed tomography revealed no abnormalities. The lesions were recalcitrant to liquid nitrogen, oral vitamin A, topical tazarotene, halobetasol, topical tofacitinib, intralesional triamcinolone, and methotrexate. Methotrexate was discontinued due to hypertriglyceridemia and diarrhea. Methotrexate rechallenge triggered a cutaneous flare, prompting pursuit of alternative therapies.

Next-generation sequencing identified a somatic KRAS (exon2, p.G12A) mutation, and cobimetinib 40 mg daily with intralesional triamcinolone was initiated. Marked flattening of lesions occurred within 2 months (Fig 1, *C* and *D*). Due to gastrointestinal toxicity, the dosage was reduced to 20 mg daily on days 1-2 and 40 mg on day 3, in a repeating cycle. However, persistent toxicity required drug discontinuation 10 months after initiation, resulting in recurrence of facial lesions. An alternative MEK inhibitor, binimetinib 60 mg daily, restored clinical response and was well tolerated. Biannual ophthalmological evaluations are performed to monitor for ocular toxicity.

## Discussion

This case describes a patient with treatment-refractory MAXG with marked response to MEK inhibition. Although few studies have evaluated the treatment of MAXG with MEK inhibitors, this report highlights their potential as a novel therapy for MAXG and related histiocytic disorders.

Our patient presented with periorbital, zygomatic, and thoracic erythematous-yellow-brown plaques. Histopathology demonstrated dense foamy histiocytes, Touton giant cells, and lymphocytic infiltrates, consistent with xanthogranulomatous dermatitis. Genetic sequencing identified a somatic KRAS (exon2, p.G12A) mutation, causing a glycine-to-alanine substitution at codon 12, which impairs GTP hydrolysis and results in constitutive downstream signaling.

The differential diagnosis encompasses other granulomatous diseases, many of which were inconsistent with the clinical presentation. The patient’s asthma predated the xanthogranulomas, and she lacked the characteristic periocular inflammatory features of adult-onset asthma with periocular xanthogranuloma. Lack of skeletal/visceral abnormalities did not support Erdheim-Chester disease. While hypercholesterolemia was present, absence of respiratory or upper gastrointestinal involvement made xanthoma disseminatum unlikely.^4^ Ultimately, multiple biopsies demonstrating xanthogranulomatous dermatitis supported a diagnosis of MAXG.

MAXG is characterized by the presence of 5 or more xanthogranuloma lesions in patients over 14 years of age. Fewer than 50 cases have been reported in the literature. MAXG is thought to arise from CD14+ dermal/interstitial dendrocytes, though the etiology remains unclear.^5^ Clinically, lesions are typically asymptomatic red-to-yellow-to-brown papules/nodules on the head and trunk. Histology reveals foamy, lipid-laden histiocytes, Touton giant cells, and mixed inflammatory infiltrates. Lesions are CD1a and S-100 negative, supporting their non-Langerhans cell origin.^5^

Recent literature suggests the xanthogranuloma family represents a continuum of disorders ranging from AXG to Erdheim-Chester disease, with overlapping histopathologic and molecular features. The patient’s shared KRAS mutation corroborates the continuum hypothesis, and her presentation likely reflects the cutaneous-predominant end of this spectrum. Other cases may harbor different mutations, most commonly BRAF V600E, or present with systemic rather than skin-limited involvement.^6^

MAXG is associated with hematologic malignancies, particularly leukemias and lymphomas, warranting monitoring; our patient’s complete blood count and serum protein electrophoresis remained unremarkable. Bone marrow biopsy was not obtained. In a study of over 4000 patients with histiocytic disorders, 118 patients had a concurrent hematologic malignancy, and 11 patients showed identical genetic mutations in both histiocytic and hematologic neoplasms, supporting a clonal relationship.^7^ No standardized malignancy screening guidelines exist due to the rarity of this condition. Nevertheless, clinicians should remain vigilant for multiple myeloma, leukemia, and lymphoma. Current recommendations include ophthalmologic evaluation, hepatic function monitoring, and complete blood counts for patients with more than 2 xanthogranulomatous lesions. Brain MRI or abdominal ultrasound is indicated if neurologic or abdominal symptoms develop.^4^

While no standard treatment exists, reported therapies include corticosteroids, retinoids, thalidomide, CO_2_ laser therapy, cryotherapy, excision, and methotrexate.^5^ MAPK pathway mutations are implicated in histiocytic disorders such as Erdheim-Chester disease and malignant histiocytosis.^8^

The identification of KRAS mutations in MAXG warrants consideration of MEK inhibitors, which suppress downstream RAS signaling, specifically inhibiting MEK-mediated ERK activation. A recent study (*N* = 29) demonstrated that 79.3% and 82.8% of MAXG skin biopsies stain positive for cyclin-D1 and phospho-ERK, respectively, which can support MAPK pathway activation when molecular testing is unavailable.^5^ Additionally, a clinical trial of 18 cobimetinib-treated patients with histiocytosis reported a response rate of 89%, with 94% of patients remaining progression-free at 1 year.^9^ Treatment duration remains unclear. Although targeted therapies demonstrate high efficacy, they often require prolonged administration due to significant risk of relapse upon discontinuation.^6^

Rosai-Dorfman disease, another non-Langerhans cell histiocytoses that may harbor KRAS mutations, has also demonstrated response to MEK inhibition.^10^ Within 2 months of MEK inhibitor therapy, our patient experienced marked lesion flattening. Discontinuation of cobimetinib resulted in recurrence, underscoring disease reactivation and the importance of maintenance therapy. This case supports a pathogenic role for somatic MAPK pathway mutations and highlights MEK inhibition as a promising treatment for MAXG and related histiocytic disorders.

## Conflicts of interest

ELD discloses unpaid editorial support from Pfizer Inc and paid advisory board activities with Day One Biopharmaceuticals and Opna Bio (outside of the submitted work).
